# Low soil Moisture Slows Uptake and Elimination Rates of Phenanthrene in Springtails

**DOI:** 10.1007/s00128-024-03854-5

**Published:** 2024-01-28

**Authors:** Yang Wang, Stine Slotsbo, Peter B. Sørensen, Martin Holmstrup

**Affiliations:** Department of Ecoscience, Aarhus University, Building 1120, C.F. Møllers Allé 4, Aarhus C, 8000 Denmark

**Keywords:** Soil water potential, Drought, Springtails, Toxicokinetics, Uptake, Elimination

## Abstract

This study investigated the influence of soil water status on the toxicokinetics of phenanthrene in the springtail *Folsomia candida* allowing estimation of uptake and elimination rates at two contrasting soil water potentials. Fitting a three-phase model to the observations showed that uptake rate (*k*_*u*_) was almost two times higher in moist soil (-2 kPa) than in dry soil (-360 kPa). During the first days of the exposure, elimination rate (*k*_*e*_) was not significantly different in moist and dry soil, but after eight days *k*_*e*_ had increased significantly more in moist soil than in dry soil. Our results confirm the general notion that the exposure route via soil pore water is important. Understanding the significance of soil moisture in exposure and effects of contaminants on soil invertebrates is crucial for assessing the ecological risks associated with soil pollution in a changing climate.

## Introduction

Soils are increasingly becoming sinks for hydrophobic organic compounds (HOCs) as a result of sewage sludge deposition, incomplete combustion of organic matter and fossil fuels and other high-temperature industrial processes (Heywood et al. 2006). Many HOCs are persistent and toxic to soil organisms and their behavior and fate in the environment are determined by their physical/chemical characteristics and the in situ conditions of soils (Jensen and Mesman 2006). Once in the soil, a fraction of the HOCs is preferentially adsorbed to organic matter and can be bound there for a long time due to their strong sorption to organic matter (Krauss and Wilcke 2001). Passive uptake of HOCs in soil invertebrates such as springtails can occur along different routes. Volatile compounds diffuse through soil pore air and rapidly enter the body across the skin (Schmidt et al. 2013). Non-volatile HOCs may be passively transported dissolved in soil pore water or bound to dissolved organic polar compounds and enter the body across the moist skin (Jager et al. 2005) or partition by direct dermal contact (Mayer and Holmstrup 2008). Lastly, HOCs may enter the body across intestinal tissues by ingestion of contaminated food particles (Ma et al. 1995).

HOCs tend to spontaneously partition into lipid-rich tissues (Endo et al. 2011). After entering tissues, cells can detoxify HOCs by catalyzing the hydrophobic structures into water-soluble forms, facilitating their degradation in cells and excretion from the body through feces or urine (Nota et al. 2009; Stroomberg et al. 2004). Many different pathways – often inducible – are responsible for the metabolism of lipophilic compounds, in which various enzymes and macromolecules play important roles in metabolism, detoxification and elimination processes (Nota et al. 2009). At steady state, bioaccumulation of HOCs results from a balance between uptake and elimination.

Drought conditions can interfere with springtails’ tolerance to HOCs, and vice versa (Sjursen and Holmstrup 2004; Sjursen et al. 2001). For example, a full-factorial 28-day mesocosm experiment showed that the EC50 for recruitment of springtails was reduced from 40 to 10 mg phenanthrene kg^− 1^ dry soil with decreasing soil water content from 22 to 12% of dry weight (equivalent to soil water potentials of -2 and − 15 kPa, respectively). However, the seemingly higher effect in dry soil was related to higher bioaccumulation in dry soil (Wang et al. 2023). In addition, exposure to dry soil resulted in low body water contents which is likely physiologically challenging (Bayley and Holmstrup 1999; Wang et al. 2022) and may decrease the detoxification capacity of springtails. Thus, external soil environmental conditions are critical to the toxicokinetics of phenanthrene in springtails. To follow up on these previous results and better understand whether the increased bioaccumulation in dry soil is due to increased uptake rates or decreased elimination rates, we performed a toxicokinetic experiment consisting of a 14-day uptake phase followed by a 6-day elimination phase exposing the springtail *Folsomia candida* to phenanthrene in soils of contrasting soil water potentials. The duration of the experiment was chosen based on previous observations indicating that steady state during the uptake phase was reached in 7–14 days, and complete elimination of phenanthrene by *F. candida* took about 5 days (Mikkelsen et al. 2019).

Here, we compared the toxicokinetic characteristics of a common HOC in moist and dry soil using *F. candida* as model organism. We used phenanthrene as a model HOC because it is widespread in the environment and is easy to measure in soil and animals (Dai et al. 2019). We assumed that the bioavailability of phenanthrene was higher in dry soil due to the hydrophobic and relatively volatile nature of phenanthrene (Schmidt et al. 2013) and therefore hypothesized that (1) the uptake rate is higher in dry soil. We assumed that insufficient body water in dry soil hampers detoxification and excretion processes and hypothesized that (2) the elimination rate is lower in dry soil. Finally, we expected that the detoxification system could be induced and enhanced during the exposure period and therefore hypothesized that (3) the elimination rate increased over time. While the previous study by Wang et al. (2023) was focused on the interactive effects of phenanthrene and soil moisture on the growth and reproduction of *F. candida* it did not provide any mechanistic understanding of the toxicokinetic aspects in these interactions. With the present study, we aim to provide this understanding.

## Materials and Methods

### Test Animals

*Folsomia candida* (Collembola, Isotomidae) originated from a laboratory culture kept at 20 °C (± 1 °C) with a 12:12 h light-dark cycle in Petri dishes with moistened plaster of Paris mixed with charcoal (8:1 w/w). The springtails were age-synchronized for the experiment, to reduce biological variation, and only medium-sized adults (25 ± 3 days old) were used.

### Phenanthrene-contaminated Soil

Organically farmed agricultural soil from the top 0–20 cm was collected in Foulum, Denmark. This soil was loamy sand consisting of 32% coarse sand (> 200 μm), 48% fine sand (20–200 μm), 9% silt (2–20 μm), 7% clay (< 2 μm), 4% organic matter (determined by loss-on-ignition), and a pH of 5.9. The total organic carbon content was 1.6%. The soil was thoroughly homogenized, dried at 105 °C for 24 h, and sieved through a 2 mm mesh before use. The soil was spiked with phenanthrene (Sigma Aldrich, CAS #85-01-8, 98 purity) dissolved in acetone (J.T. Barker, HPLC quality) using 180 mL kg^− 1^ dry soil. The solution and dry soil were thoroughly mixed to obtain a sublethal phenanthrene concentration of 40 mg kg^− 1^ dry soil (Wang et al. 2023). The spiked soil was left overnight under a fume hood to allow the acetone to evaporate. The actual phenanthrene concentrations in the soil were measured using GC‒MS (see later description) and showed fairly good agreement between the nominal and actual concentrations in the test soil (Supplementary Fig. S1). Field realistic concentrations of total HOCs may reach 10 − 20 mg kg^− 1^ dry soil in industrialized areas of temperate regions (Jiao et al. 2017; Sun et al. 2018). Thus, the concentration of phenanthrene of the present research was quite high but used to investigate principles behind the roles of soil moisture for toxico-kinetics.

### Soil Moisture

Soil water contents were adjusted at 6 and 20% of dry weight by adding appropriate volumes of deionized water to the spiked soil and thoroughly mixed. The actual soil water content was calculated by subtracting the soil weights before and after drying at 105 °C for 24 h and showed that the measured water content was 6.05 ± 0.08 and 20.32 ± 0.43% of dry weight, respectively. These soil water contents corresponded to -357 ± 59 kPa and − 2.4 ± 0.05 kPa, respectively (Wang et al. 2022).

### Toxicokinetics Experiment Setup

To examine phenanthrene concentrations in springtails over time, ten animals were added to 150 mL jars containing 30 g spiked soil as previously described (Wang et al. 2023). In total, 60 jars were set up for each soil water content (12 sampling times, 5 replicates for each sampling time). To describe the toxicokinetics, we collected springtails during the first 14 days (days 0, 1, 2, 3, 5, 8, 10, 14) to describe the uptake and equilibrium phases. After 14 days, the springtails of each remaining jar were collected and transferred to similar jars containing uncontaminated soil of the same water content. Animals were then sampled over the following 6-day period at four occasions (day 15, 16, 17 and 20) to describe the elimination phase. Soil (sampled at days 0, 8 and 14) and animal samples were stored at -80 °C for further analysis. During the experiment, water evaporation from the beakers was negligible because the lids were only briefly opened for aeration every 3 days. Two milligrams of dried yeast were added to the soil surface in each beaker and replenished every 7 days as the only food resource for the springtails.

### Sampling of Animals for Survival and Adult Fresh Weight

The soil and animals in each beaker were gently emptied into a tray. All adults in each beaker were collected using an aspirator, and their combined fresh weight was determined using a Sartorius Micro SC 2 balance accurate to ± 1 µg (Sartorius AG, Goettingen, Germany). The number of surviving adults was counted. The animals were placed in 2 ml microcentrifuge tubes and snap-frozen at -80 °C. The animal harvesting procedure from collection to freezing took approximately 5–8 min per sample.

### Determination of Body Water Content in Moist and Dry Soil

In order to determine the development of body water content in moist and dry soil, ten springtails per jar containing 30 g of non-contaminated soil were exposed for up to 20 days. In total, 60 jars were set up for each soil water content (12 sampling times, 5 replicates). These adults were collected, and their combined fresh weights were determined as described above. Their combined dry weight was determined by weighing after freeze-drying for 24 h and body water content was calculated as mg water mg^− 1^ dry weight (Supplementary Fig. S2). we converted the fresh weight of phenanthrene-exposed adult springtails to dry weight and calculated an internal concentration of phenanthrene on a dry weight basis.

### Determination of Phenanthrene Content in Adult Springtails and soil

Since soil water content influences the springtails’ body water content (Supplementary Fig. S2) we had to estimate the internal concentration of phenanthrene on a dry weight basis as described by Wang et al. (2023) using the linear relationship between exposure time and the water content of springtails exposed to noncontaminated soil (Supplementary Fig. S2) to transform the fresh weight of phenanthrene-exposed springtails to dry weight and calculate an internal concentration of phenanthrene on a dry weight basis.

Phenanthrene concentrations in animal tissues were measured according to the method described by Holmstrup et al. (Holmstrup et al., 2014). In brief, the adults from each replicate were transferred to a 1.5 mL brown glass vial, and 500 µL of acetonitrile (VMR international, USA) was added. The vials were placed in a sonicator (Thermo, Germany), sonicated for 90 min on ice, kept at room temperature for 24 h, frozen at -18 °C for 24 h and kept at room temperature for another 24 h. The samples were sonicated again for 90 min on ice and then transferred to 1.5 mL tubes for brief centrifugation (3 min at 2,400 g). The supernatant from each tube was transferred to an autosampler vial and stored at -80 °C until phenanthrene analysis by GC‒MS (GCMS-QP2010, Shimadzu, Japan). Phenanthrene standards, including blanks, were run in parallel and subjected to the same extraction procedure.

Phenanthrene in soil samples (1 g fresh weight) was extracted with 4 mL of acetonitrile by shaking at 200 rpm for 24 h, followed by centrifugation at 1000×*g* for 5 min. The supernatants were transferred to autosampler vials and analysed as described above. For quality control, blank medium and uncontaminated soil were analyzed using the same procedures.

The limit of detection (LoD) and the limit of quantification (LoQ) of phenanthrene in animals were 4.5 − 10.5 and 16.5 − 37.8 mg phenanthrene/kg dry weight, respectively. The LoD and LoQ of phenanthrene in soil were 0.11 and 0.36 mg phenanthrene/kg dry soil, respectively. Recovery was tested by spiking uncontaminated animal material with known amounts of phenanthrene and ranged between 93.2 and 108.4%, with an average (± standard deviation) of 101 ± 6%.

### Uptake and Elimination Modeling

The degradation of phenanthrene in the soil was based on a linear regression using data for days 0, 8 and 14 (Supplementary Fig. S1)

1 $${C_s} = a \cdot t + {C_{si}}$$

where ${C}_{s}$ is the concentration of phenanthrene in soil at time *t* (g/kg dw), ${C}_{si} (t=0)$ is the initial concentration of phenanthrene in the soil, and *a* is the rate of phenanthrene degradation in soil.

The kinetics of phenanthrene concentrations in springtail tissues were described by a three-phase model. Extremely low concentrations of phenanthrene in animal tissues on days 17 and 20 prevented proper modeling of toxicokinetics from fitting the raw data, and hence, data for these days were not included in the parameter estimation. The three-phase model was as follows:

Uptake from soil into the organism:

2 $$\frac{d{C}_{int}}{dt}=Uptake-Elimination$$

where ${C}_{int}$ is the concentration of phenanthrene in springtails [g/kg dw]. The uptake is assumed to follow the first-order kinetic relation:

3 $$Uptake={k}_{u} \cdot {C}_{s}$$

where ${C}_{s}$ is the soil concentration of phenanthrene at time *t* [g/kg dw] and ${k}_{u}$ is the uptake rate coefficient from soil into the organism (per day). The elimination is described by the first-order kinetic relation:

4 $$Elimination={k}_{e} \cdot {C}_{int}$$

where ${k}_{e}$ is the elimination rate coefficient (per day), which is assumed to be composed of two rate coefficients: (1) an initial elimination rate coefficient (${k}_{ei}$) and a later elimination rate coefficient after further activation of the detoxification system (${k}_{ex}$). Hence, the elimination coefficient is described as

$${k}_{e}={k}_{ei}+{\Delta }{k}_{e} \cdot N(t,{T}_{t},\sigma$$) (5)

where N() is the accumulated normal distribution, ${T}_{t}$ is the time of transition between ${k}_{ei}$ and ${k}_{ex}$ [d], and $\sigma$ is a characteristic time value [d] for the duration of the transition between a condition where ${k}_{e}\approx {k}_{ei}$ ($t\ll {T}_{t} =>N(t,{T}_{t},\sigma )\approx 0)$to the later condition where ${k}_{e}\approx {k}_{ex}$ ($t\gg {T}_{t} =>N(t,{T}_{t},\sigma )\approx 1). \text{T}\text{h}\text{e} \text{t}\text{r}\text{a}\text{n}\text{s}\text{i}\text{t}\text{i}\text{o}\text{n}$is assumed to be relatively fast, so the value of $\sigma$ is fixed to be 0.1 d. ${{\Delta }{k}_{e}=k}_{ex}-{k}_{ei}$ is the difference in elimination rates.

Thus, for each soil moisture (6 and 20%), the model needs to estimate ${k}_{u}$, ${k}_{e}$, ${k}_{ei}$ and ${T}_{t}$.

BAF is described as

6 $$\text{B}\text{A}\text{F}=\frac{{k}_{u}}{{k}_{e}}$$

Statistical significance of the effect of soil moisture on uptake and elimination constants were tested using the Wilks Theorem for the Log Likelihood Test. The residual distribution between model predictions and measured concentration levels was assumed to be Log Normal, and based on this the Log likelihood was estimated as input to the Log likelihood testing (Wilks 1938).

## Results

The survival proportion at 6% soil water content was approximately 90% over the entire experiment (Supplementary Fig. S3, *p* < 0.001), but no significant decrease in survival was observed at 20% soil water content.

**Fig. 1:**
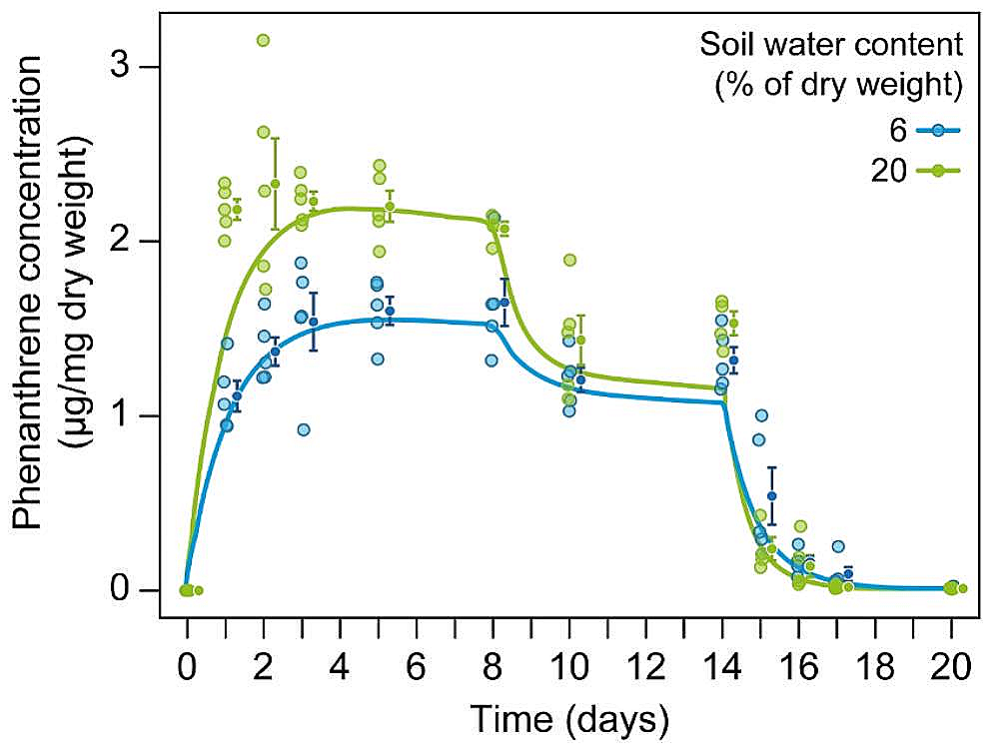
The concentration of phenanthrene in springtail tissues during uptake (days 0–14; 40 mg/kg dry soil) and in the elimination phase (days 14–20; uncontaminated soil). Lines represent model fitting to raw data. Dots represent observations in each group for each day. Points with error bars represent the mean ± SE (*n* = 3–5)

A three-phase toxicokinetic model, including phenanthrene degradation in soil, generally fitted the data well at both soil moistures (R^2^_20%_ = 0.81 and R^2^_6%_ = 0.88; Fig. 1 and Supplementary Table S1). In the initial uptake phase, the internal phenanthrene concentration increased significantly faster at 20% moisture (${k}_{u}$= 62) than at 6% soil moisture, where it gradually climbed to a steady state in 3 days (${k}_{u}$= 38, *p* = 0.001; Table S1). After the initial uptake, the concentration of phenanthrene in springtails had reached the first steady state after 5–8 days (S_1_) in both dry soil and humid soil (S_1_ = 1.54 and 2.17 g kg^− 1^ dw, respectively; Table S1). Between days 8 and 10 the internal concentration decreased by 30% to 1.09 g kg^− 1^ dw in the dry soil, and by 46% to 1.18 g kg^− 1^ dw in humid soil reaching a second steady state (S_2_) until shifting to the elimination phase in uncontaminated soil (Fig. 1; Table S1).

When the springtails were transferred to uncontaminated soil, rapid elimination was observed, and within 48 h, 96% of phenanthrene was eliminated from animal tissue in the 20% soil water content group and 89.7% in the 6% soil water content group (Fig. 1). The initial elimination rate constant ($${k}_{ei})$$ was not significantly different at 20% (0.84) and 6% (0.94) soil water content (Table S1). However, the elimination constant after 8 days ($${k}_{ex})$$ had increased to 1.56 and 1.12 at 20% and 6% soil water content, respectively (Table S1). Thus, we observed that$${\Delta }{k}_{e}$$ at 20% soil water content (0.62) was two times higher than that at 6% soil water content (0.28) (*p* < 0.001, Table S1).

## Discussion

Although soil moisture is perhaps the most important environmental factor for performance of soil organisms, and in particular soil invertebrates (Blankinship et al. 2011), the significance of this factor for uptake and elimination rates of organic contaminants has, surprisingly, not previously received any attention from scientists. We show, for the first time, that soil water content has dramatic effects on both uptake and elimination rates. This new information complements our previous study on the interaction between effects of soil moisture and HOCs such as phenanthrene (Wang et al. 2023), and shows that the effects manifested in life-history traits such as growth and reproduction are significantly influenced by exposure (toxico-kinetics), and not only due to cellular and molecular toxicity effects.

Contrary to our first hypothesis stating that *k*_*u*_ would be higher in dry soil than in moist soil, *k*_*u*_ was almost twice as high in the moist soil. This suggests that the exposure route via soil pore water is significant and facilitates uptake. It may also reflect that the springtails were more active in moist soil (data not shown) and therefore had increased contact with contaminated soil particles or higher ingestion of contaminated organic matter in the moist soil compared to the dry soil. A few studies on organic contaminant toxicity have accounted explicitly for solubility and soil pore water concentration of HOCs (Jager et al. 2003; Krauss and Wilcke 2001). The fast uptake of phenanthrene in this study is consistent with previous studies demonstrating that rapid uptake of PAHs (phenanthrene and pyrene) takes place in springtails (Mikkelsen et al. 2019; Schmidt et al. 2013), suggesting that passive transport of HOC in the pore water can be influenced by the kinetics of contaminant desorption and mass transport. Krauss and Wilcke (2001) stated that the amounts of HOC combined with dissolved ligands in water are often higher than those of free-diffusing dissolved HOC. This implies that most water-borne HOC uptake is mediated through ligand binding rather than free HOC diffusing directly into animals.

Based on the hydrophobic properties of HOCs, a study by Jager et al. (2000) supported that the uptake constants for PAHs in artificial soil medium showed a clear decrease with increasing K_ow_ for phenanthrene, pyrene, fluoranthene and benzo[a]pyrene, however, this relationship disappeared when *k*_*u*_ was based on pore water concentrations, indicating that water facilitated HOC transport as the main route. The role of soil pore water is perhaps more direct for charged molecules, such as metal ions that are readily dissolved in water. For example, the uptake rate of Cd in earthworms was lower in dry soil than in moist soil, suggesting that metal absorption also depends on soil water content (Gonzalez-Alcaraz et al. 2018).

Considering that the soil water potential is critical to springtails’ physiological status and capacity (Bayley and Holmstrup 1999; Wang et al. 2022), our second hypothesis assumed that *k*_*e*_ was lower in dry soil due to insufficient body water for detoxification and excretion processes (Wang et al. 2023). We observed that springtails when exposed to dry soil suffered from dehydration, and that *k*_*e*_, as a critical physiological indicator, showed that springtails could not detoxify phenanthrene as efficiently in the dry soil as in moist soil, confirming our second hypothesis. This was maybe because detoxification of phenanthrene depends on the animal’s body water status and energy supply for transforming compounds from being hydrophobic to more hydrophilic, facilitating excretion through urine and feces. Thus, dehydration of springtails could lower their urine secretion, leading to reduced phenanthrene detoxification and excretion.

Testing our third hypothesis, we confirmed that the detoxification system was induced and enhanced during the exposure period. Hence, the elimination rate increased over time both in moist and in dry soil, but Δ*k*_*e*_ was two times higher in humid soil than in dry soil. In moist soil, Δ*k*_*e*_ showed that the detoxification of phenanthrene in springtails was boosted efficiently, probably due to sufficient body water content and energy supply for the transformation of HOC into water-soluble forms. Induction of detoxification systems has also been observed in earthworms. For example, first-order kinetic two-compartment modeling of phenanthrene uptake showed, in a peak shape, that concentrations in earthworms rapidly reached maximum levels and then decreased by 50% over 14 d (Zhang et al. 2017). Similarly, using a three-phase kinetics model, the internal concentration of 4-nonylphenol in worm tissue was also peak-shaped in the uptake phase, which reached the maximum concentration on the first day and then decreased by approximately 50% over the next 4 d to reach a new steady state (Patricio Silva et al. 2016). These reports suggest that animals can actively increase their detoxification capacity, but mostly so under benign environmental conditions.

Based on the estimated parameters of the modelled uptake and elimination rate constants we found that BAF of phenanthrene in moist soil decreased from 66 at the initial steady state to 40 after two weeks of exposure (second steady state). In dry soil BAF decreased from 45 to 34. These values of BAF are in good agreement with previous observations using the same springtail species and soil type (Wang et al. 2023). However, in the present study we saw that low soil moisture resulted in slightly lower BAF than in moist soil (34 vs. 40) when assessed after 2 weeks of exposure, whereas in our previous study we observed *higher* BAF in 6% soil water content than in 20% soil water content (60 vs. 40) after 4 weeks of exposure (Wang et al. 2023). This suggests that exposure time has an impact on the effect of soil moisture on BAF.

In soil, carrier-mediated transport in pore water is important for HOC partitioning. Drought causes the soil water to retract to so small capillary pores that liquid water becomes practically unavailable to springtails, and furthermore disrupts the important carrier-mediated transport of HOCs in water to animal tissues. Additionally, drought stress leads to decreased locomotory behavior, dehydration, starvation and reduced detoxification. Functioning of ecosystems is threatened by the impacts of multiple stressors, e.g., rising temperatures, toxic pollutants, and drought, all at the same time, so the combined effects of these stressors are hard to predict. Given that soil moisture is dynamic in time and space, as is the distribution of pollutants, this study contributes to a better understanding of the toxicokinetics of HOCs in invertebrates in a changing environment.
